# The *Dictyostelium *genome encodes numerous RasGEFs with multiple biological roles

**DOI:** 10.1186/gb-2005-6-8-r68

**Published:** 2005-07-28

**Authors:** Andrew Wilkins, Karol Szafranski, Derek J Fraser, Deenadayalan Bakthavatsalam, Rolf Müller, Paul R Fisher, Gernot Glöckner, Ludwig Eichinger, Angelika A Noegel, Robert H Insall

**Affiliations:** 1 School of Biosciences, University of Birmingham, Birmingham B15 2TT, UK; 2 Genome Analysis, Institute for Molecular Biotechnology, Beutenbergstrasse 11, D-07745 Jena, Germany; 3 Department of Microbiology, La Trobe University, VIC 3086, Australia; 4 Centre for Biochemistry and Centre for Molecular Medicine Cologne, Medical Faculty, University of Cologne, Joseph-Stelzmann-Strasse 52, 50931 Cologne, Germany

## Abstract

**Background:**

*Dictyostelium discoideum *is a eukaryote with a simple lifestyle and a relatively small genome whose sequence has been fully determined. It is widely used for studies on cell signaling, movement and multicellular development. Ras guanine-nucleotide exchange factors (RasGEFs) are the proteins that activate Ras and thus lie near the top of many signaling pathways. They are particularly important for signaling in development and chemotaxis in many organisms, including *Dictyostelium*.

**Results:**

We have searched the genome for sequences encoding RasGEFs. Despite its relative simplicity, we find that the *Dictyostelium *genome encodes at least 25 RasGEFs, with a few other genes encoding only parts of the RasGEF consensus domains. All appear to be expressed at some point in development. The 25 genes include a wide variety of domain structures, most of which have not been seen in other organisms. The LisH domain, which is associated with microtubule binding, is seen particularly frequently; other domains that confer interactions with the cytoskeleton are also common. Disruption of a sample of the novel genes reveals that many have clear phenotypes, including altered morphology and defects in chemotaxis, slug phototaxis and thermotaxis.

**Conclusion:**

These results suggest that the unexpectedly large number of RasGEF genes reflects an evolutionary expansion of the range of Ras signaling rather than functional redundancy or the presence of multiple pseudogenes.

## Abstract 2

A survey of the *Dictyostelium *genome reveals at least 25 RasGEFs, all of which appear to be expressed at some point in development. Disruption of several of these novel RasGEFs reveals that many have clear phenotypes, suggesting that the unexpectedly large number of RasGEF genes reflects an evolutionary expansion of the range of Ras signaling.

## Background

Ras proteins are small GTPases that sit at the center of numerous signaling pathways in essentially all eukaryotes [1]. Their activity is controlled by which guanine nucleotide is bound. When it is GDP, the Ras proteins are inactive and do not bind to their targets. Guanine-nucleotide exchange factors (RasGEFs) catalyze the replacement of GDP with GTP [2]. This makes the Ras proteins active, and able to bind multiple activators and signal transducers. RasGEFs are therefore the initiators of Ras signaling, and understanding their behavior is the key to understanding Ras signaling.

### Multiple roles of Ras pathways

The *RAS *gene was originally described as the cellular counterpart of a viral oncogene, v-*ras *[3]. The virally encoded protein, which is constitutively GTP-bound even in the absence of RasGEFs and is therefore constantly active [4], causes unchecked mitogenesis and proliferation in appropriate cell lines. Normal mammalian cells encode three different Ras families, Ha-Ras, Ki-Ras and N-Ras, all members of which are highly similar to one another. Examination of tumors from numerous patients has since confirmed that endogenous Ras has a key role in growth control - as many as 90% of pancreatic carcinomas contain a mutated *Ras *gene similar to v-*ras*.

The connection between Ras and growth has now been found to be far more complex. In primary cultures, expression of activated Ras causes apoptosis, not unrestricted growth, and activation of Ras has been shown to cause a range of effects including increased cell motility [5], macropinocytosis [6], and alterations in cell identity [7]. These changes are mediated by a range of downstream effectors, most important of which are the lipid kinase phosphatidylinositol 3-kinase (PI3K) and the protein kinase Raf [8,9].

RasGEFs were first identified in *Saccharomyces cerevisiae*, in which loss of the *CDC25 *gene was found to arrest growth by blocking Ras activation of adenylyl cyclase [10]. This was followed by the identification of *Drosophila *Son of sevenless (Sos) [11] and mammalian hSos1 [12], each of which contains a catalytic domain related to that in CDC25. Hundreds of RasGEFs are now known. All share a considerable stretch of homology, including at least two discrete domains - an amino-terminal domain of unclear function (although crystallographic evidence suggests a structural role [13]) and a carboxy-terminal one that mediates GTP-GDP exchange.

### RasGEFs and signaling

In general, RasGEFs are now seen as signaling adaptors and integrators; they couple various signaling processes at the cell membrane to Ras and thus to changes inside the cell. The best understood signals to Ras derive from receptor tyrosine kinases (RTKs). When RTKs are stimulated by their ligands, they recruit adaptors such as Grb2 [14], which bind directly to RasGEFs. This recruitment localizes the RasGEFs to the membrane and thus brings them into proximity with Ras [15]. Other RasGEFs are activated by different signals, for example Ca^2+ ^[16], but the underlying mechanism is thought to be similar. The domains that surround the RasGEF catalytic regions are therefore critical, as they mediate membrane localization and activation.

Several major families of RasGEFs can be found in the literature, classified by their domain structure. The most widely known is typified by the product of the *Drosophila Sos *gene [11]. Members contain one or two pleckstrin homology (PH) domains, implying upstream regulation by membrane phospholipids. They also contain a Dbl homology (DH) domain, which is also found in GEFs for the small GTPases Rho and Rac, although these domains' association with Rac signaling is far less clear than their proven roles as Ras regulators [17]. The EPAC family of GEFs contains cyclic nucleotide monophosphate binding domain (cNMP) motifs that bind cAMP and activate the GTPase Rap when cAMP is present [18]. Similarly, the Ras-GRF family of RasGEFs contain EF hands and activate Ras in response to calcium and diacylglycerol signaling [16]. A significant number of known RasGEF relatives have no obvious signaling domains. The best known of these is C3G, which is thought to activate the Ras-like GTPase Rap1 [19].

### Ras pathways in *Dictyostelium*

The social ameba *Dictyostelium discoideum *uses Ras pathways to control multiple signaling processes including cell movement, polarity and cytokinesis, chemotaxis, macropinocytosis and multicellular development [20,21]. It is notable for the relatively large number of Ras subfamily members (there are 15 encoded in the genome, including 11 that most closely resemble Ras, three Rap and one Rheb [22]). All of the six so far studied appear to have nonredundant and important roles in cell physiology [23-26] (although six of the remaining *ras *genes are exceptionally similar [22]). This is the more surprising as *Dictyostelium *does not encode RTKs [27]. As described previously, RTKs are thought to be one of the major inputs for Ras signaling in mammalian cells. In the absence of RTKs, the best prospects for finding upstream regulators would appear to be through identification of adaptor proteins and binding partners. Such proteins are presumably responsible for recruiting RasGEFs to the membrane and thus controlling their activity. However, two of the four *Dictyostelium *RasGEFs characterized thus far (*aimless *[28] and *rasgefB *[29]) offer few clues to their regulation. Unlike nearly all other RasGEFs described in the literature, which contain a panoply of protein-interaction and regulatory domains including SH3, PH, IQ, and PDZ domains, neither Aimless nor RasGEFB contains recognizable signaling domains [28,29]. Two other RasGEF family members, GbpC and GbpD [30], on the other hand, contain multiple domains, including cGMP-binding domains, and in GbpC, a kinase domain, leucine-rich repeats, and a DEP domain. None of these domains have been shown to regulate GEF activity in *Dictyostelium*, although in the mammalian EPAC family a cAMP-binding cNMP domain is thought to regulate GEF activity [31]. It therefore seems plausible that cGMP regulates the activity of at least GbpC. The limited number of signaling domains in the other RasGEFs has been a serious obstacle to understanding Ras family signaling in *Dictyostelium*; to date, no binding proteins or signaling partners have been discovered.

In this paper we describe an unexpectedly large number - at least 25 - of predicted RasGEFs in the *Dictyostelium *genome. Several of these contain different known signaling domains and others contain none. This suggests an unprecedentedly complex and poorly understood network of Ras signaling in an organism whose signaling otherwise appears relatively simple.

## Results

### Identification of RasGEF genes

The assembly of the *Dictyostelium *genome is now complete, representing more than 99% of the genes [27]. This allowed us to estimate the total number of RasGEF sequences. We searched the assembled sequence using FASTA and TBLASTN programs and the RasGEF catalytic sequence (also known as the RasGEF domain) as bait. Approximately 30 sequences gave significant scores with these programs.

An additional domain, known as RasGEF_N (hereafter called RasGEF amino-terminal for clarity), is found in nearly all 'true' RasGEFs (proteins that clearly activate Ras *in vivo*) as well as in GEFs for a number of related proteins such as Rap1. After excluding sequences that did not contain complete copies of both RasGEF and RasGEF amino-terminal domains, we were left with 25 sequences encoding *Dictyostelium *RasGEFs. There were no obvious pseudogenes - all 25 sequences contain a clear open reading frame (ORF) and both domains were complete and uninterrupted.

Four of the RasGEF genes we found have been described previously. The *aimless *and *rasgefB *genes have roles in chemotaxis and endocytosis, respectively [28,29], while the *gbpC *and *gbpD *genes encode cGMP-binding proteins which are thought to couple intracellular cGMP to other signaling pathways [30]. For the sake of consistency, we have renamed these genes *gefA*, *gefB*, *gefT *and *gefU*.

Four further genes encode the carboxy-terminal RasGEF homology domain but not the RasGEF amino-terminal domain, and might therefore be expected to be specific for Ras-related proteins which are not part of the Ras family proper. We have named these *gflA*, *gflB*, *gflC *and *gflD *(full details can be found in Dictybase [32]). It is not yet clear whether these are likely to be RasGEFs with a subset of normal functions or GEFs for more distant relatives of Ras.

### Sequence and domain analysis

**Figure 1:**
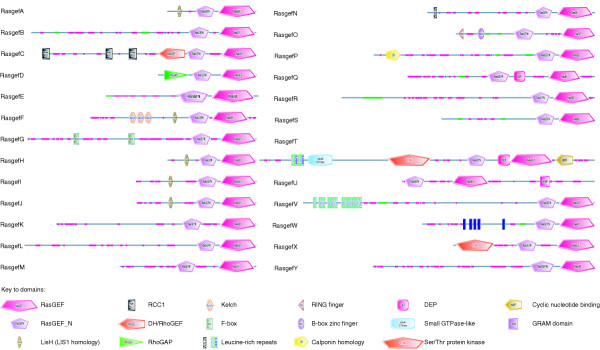
Domain structure of predicted *Dictyostelium *RasGEFs. Predicted protein sequences of all complete RasGEFs predicted from the *Dictyostelium *genome were searched in the SMART 4.0 database [34]. Individual domains are labeled in the figure. Pink, segments of low compositional complexity determined by the *SEG *program. Green, potential coiled-coil regions determined by the Coils2 program. Blue bars, transmembrane segments as predicted by the TMHMM2 program.

As described earlier, the presence of signaling domains separate from the RasGEF and RasGEF amino-terminal domain has been a central part of the identification of Ras signaling pathways in higher eukaryotes [33]. However, half of the 25 genes we have identified contain no such clues. Figure 1 shows the domain structure of the predicted products from the 25 complete RasGEFs. Eight, including RasGEFB, contain no domains detected by SMART [34] or PFAM [35], other than the two RasGEF domains. A further five contain no additional domains other than the enigmatic LisH domain, whose function is thought to relate to motility and microtubule function but is poorly understood [36], and one contains only two F-boxes, motifs connected with ubiquitination and protein breakdown rather than with upstream signaling.

We were surprised to find that none of the *Dictyostelium *RasGEFs resembled members of known families with signaling motifs. Only one of the 25 predicted proteins, GefC, includes a DH/Rho-GEF domain like *Sos *family members [17]. However, unlike the *Sos *family, GefC does not contain a PH domain. Instead, its amino-terminal region contains three domains that resemble RCC1, a GEF for the small GTPase Ran [37]. Ran is involved in the control of nuclear transport and mitosis, and is unusual in that it does not contain lipid adducts and is therefore not located at the plasma membrane [38]. There is no clear precedent for a connection between Ras and RCC1 signaling.

The two previously described cGMP-binding proteins RasGEFT and RasGEFU (also known as cGMP-binding proteins C and D [30]) have cNMP domains, like the mammalian EPAC family. RasGEFU is somewhat similar to EPAC family members, but the three cNMP domains lie beyond the RasGEF amino-terminal and RasGEF domains, unlike the usual upstream location, and the G-protein-associated DEP domain is replaced by a GRAM membrane-localization domain. These large-scale changes make it seem unlikely that EPACs and *Dictyostelium *cGMP-binding RasGEFs are evolutionary orthologs. It seems more likely that convergent evolution has selected independent cyclic nucleotide-regulated GEFs. This is supported by phylogenetic analysis (see below), which failed to group RasGEFT and RasGEFU with human EPAC.

The third principal family of mammalian RasGEFs is the calcium-regulated Ras-GRFs [16]. These do not appear to be present in *Dictyostelium *at all. Neither the EF hands present in Ras-GRFs nor any other clear calcium-binding motifs are found in any of the 25 RasGEFs examined here.

The only protein described in the literature that resembles the *Dictyostelium *RasGEF homologs is the C3G RapGEF [19], but as this similarity is based on an absence of other defined signaling domains rather than any positive homology, it seems uninformative. In particular, the *Dictyostelium *gene products lack SH3-binding polyproline domains at their amino termini, which suggests that they have no strong evolutionary relationship with C3G. Again, phylogenetic analysis supports this view (see below).

### Actin- and Rho-binding RasGEFs

Three members of the *Dictyostelium *RasGEF family have domains that suggest a direct link with the actin cytoskeleton. Two contain domains that are associated with direct binding to F-actin. RasGEFF contains three tandem kelch repeats, while RasGEFP contains a calponin homology (CH) domain. A third, RasGEFD, contains a RhoGAP homology domain. While most authors have associated this with inactivation of Rac- and Rho-family members, there is some evidence that some RhoGAP homology domains are found in downstream effectors. We were intrigued to note that *Saccharomyces *BEM2, a RhoGAP homolog that is required for cell polarity and normal actin dynamics [39], also appears to contain RasGEF and RasGEF amino-terminal domains that have not been described in the literature. This suggests that RasGEFD might perform a similar role in *Dictyostelium *to BEM2 in *Saccharomyces*, although no obvious phenotype was seen in growing *gefD *knockouts (see below).

### Phylogenetic analysis

**Figure 2:**
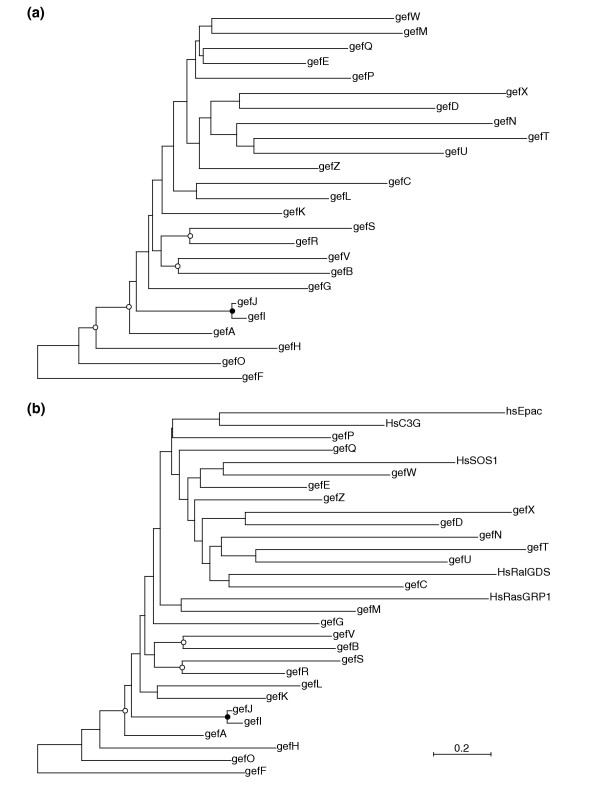
Phylogenetic analysis of predicted *Dictyostelium *RasGEFs. The RasGEF domains of **(a) **the predicted *Dictyostelium *RasGEFs alone or **(b) **the predicted *Dictyostelium *RasGEFs with selected representatives of human RasGEF families were aligned using ClustalX 1.83. Domain boundaries were predicted by SMART [34]. Phylogenetic trees were constructed using 1,000 bootstraps, excluding all matches within gaps. Closed circles indicate nodes found in 100% of bootstraps; open circles indicate nodes found in ≥50% of bootstraps. All other nodes are found in <50% of bootstraps.

We constructed phylogenetic trees using the conserved RasGEF domains of all the *Dictyostelium *proteins, both with and without a selection of mammalian RasGEFs for comparison (Figure 2). To our surprise, few groupings were strongly supported during bootstrapping. In general, it is clear that RasGEFF, RasGEFO and RasGEFH are the least similar to other RasGEFs in *Dictyostelium *and elsewhere. RasGEFI and RasGEFJ are highly similar, suggesting they they arose from a relatively recent gene duplication. Two other pairings, RasGEFs B and V and RasGEFs R and S, were also supported in more than 50% of bootstraps. Other, larger groups that might subdivide the RasGEFs according to function were conspicuously poorly supported, suggesting that the diversification of RasGEF genes happened relatively early in the divergence of *Dictyostelium*. There is also no evidence that the division of mammalian RasGEFs into SOS, RasGRP, EPAC, C3G and RalGDS families had occurred when *Dictyostelium *diverged from the animal line.

### Expression during growth and development

To determine whether the large number of *Dictyostelium *RasGEFs was connected with growth (the first role found for Ras pathways), or signaling during development, we determined the expression patterns of all of the 25 genes during growth and development. Initially we screened Northern blots; if a clear result was not obtained we used RT-PCR. We used probes generated from those clones represented in the Tsukuba cDNA project, and used PCR to make the remainder. Table 1 shows the results. All RasGEF genes are expressed at some stage in development; of the genes tested, *gefG*, of which transcripts could only be detected at 12 and 14 hours of development, was the most weakly expressed. This clearly implies that the large RasGEF family is not made up of pseudogenes or evolutionary relics, which are frequently not expressed.

In general, three patterns of expression were seen. The largest group of genes, which includes *gefA*, *D*, *F*, *H*, *J *and *L*, is expressed in growing cells and with relatively slight changes throughout development. A second group, comprising *gefB *and *K*, is expressed at low levels during growth with a sharp increase early in development, while the third, typified by *gefC *and *E*, is only expressed after about 12 h of development. These results are consistent with varied roles for RasGEFs in multiple aspects of the *Dictyostelium *life cycle.

### Disruption of a sample of RasGEF genes

There are two obvious ways to explain the unexpectedly large number of RasGEF genes in *Dictyostelium*. The first would be that these genes are mainly redundant, and that each biological function of a RasGEF is mediated by several *Dictyostelium *genes. The other possibility is that *Dictyostelium *has greatly broadened the scope of Ras signaling at some stage in its evolution, and that most GEFs are required for a distinct signaling process. To distinguish between these possibilities, we disrupted a sample of RasGEF genes and searched for phenotypes. *gefA *(*aimless*) had already been disrupted [40] and *gefB *was disrupted during the initial part of this work [41]; each mutant has a clear phenotype. We further attempted to disrupt GEFs C, D, E, F, G, K and L. We were successful in all cases except *gefF*, which could not be disrupted even after several attempts. This suggests that *gefF *might have an important role during growth, but confirmation will require disruption in diploids [42].

### Phenotypes of mutants

**Figure 3:**
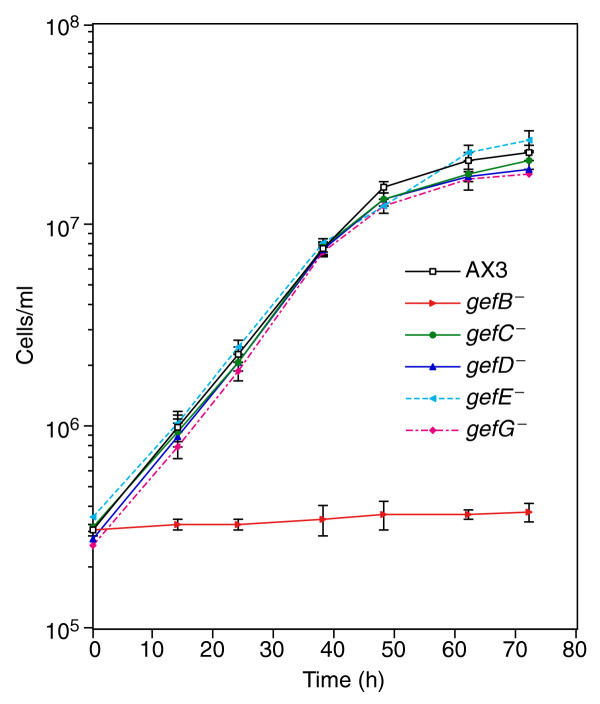
Growth of RasGEF mutants in axenic suspension. Vegetative AX3 and *gef *null cells were grown in axenic medium in shaken flasks. At the indicated times, samples were taken and counted in duplicate using a hemocytometer. Mutants in *gef*K and *gef*L were observed on different occasions but were similarly comparable to the parental strain.

**Figure 4:**
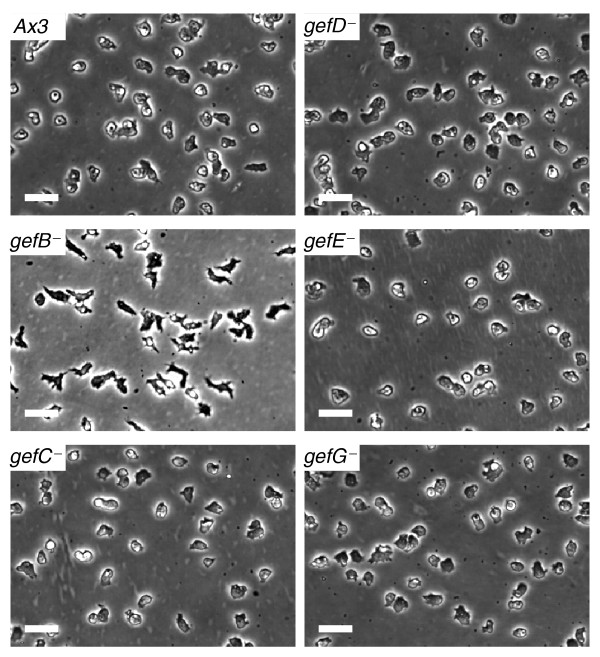
Morphology of *gef *mutant cells. Vegetative AX3 and *gef *null cells were grown in axenic medium in tissue culture dishes for 24 h and visualized by phase-contrast microscopy. The white bar represents 20 μm. Mutants in *gefK *and *gefL *were observed on different occasions but were similarly comparable to the parental strain.

**Figure 5:**
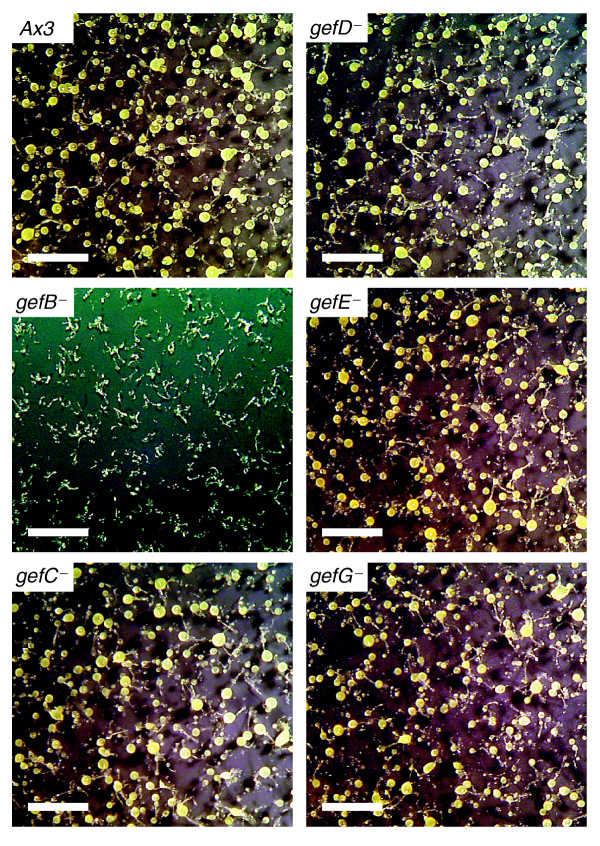
Morphology of *gef *mutant fruiting bodies. AX3 and *gef *null cells were developed at 3 × 10^6 ^cells/cm^2 ^on nitrocellulose filters for 48 h then photographed using a dissecting microscope. The white bar represents 2 mm. Mutants in *gef*K and *gef*L were observed on different occasions but were similarly comparable to the parental strain.

All of the mutants we obtained grow normally, with the exception of *gefB *mutants, which grow relatively normally on bacterial plates but are unable to grow in axenic culture due to a loss of fluid-phase endocytosis (Figure 3). The morphology of growing cells is apparently normal for each strain apart from *gefB *mutants, which appear flattened and polar (Figure 4) in a manner reminiscent of growing nonaxenic cells. Similarly, the chemotactic aggregation and development of all mutants examined here except *gefB *were apparently normal (Figure 5), forming morphologically normal slugs and fruiting bodies with the usual timing. As previously described, *gefB *mutants aggregate but form no slugs and make highly aberrant fruiting bodies [41]. Three RasGEF mutants described elsewhere show abnormal chemotaxis - *gefA*/*aimless *and *gefT *are both seriously defective [28,43], though *gefA *can be coaxed to make rather aberrantly shaped slugs, while *gefU *cells are hyperpolar and better at chemotaxis than wild type [43].

### Slug movement phenotypes

**Figure 6:**
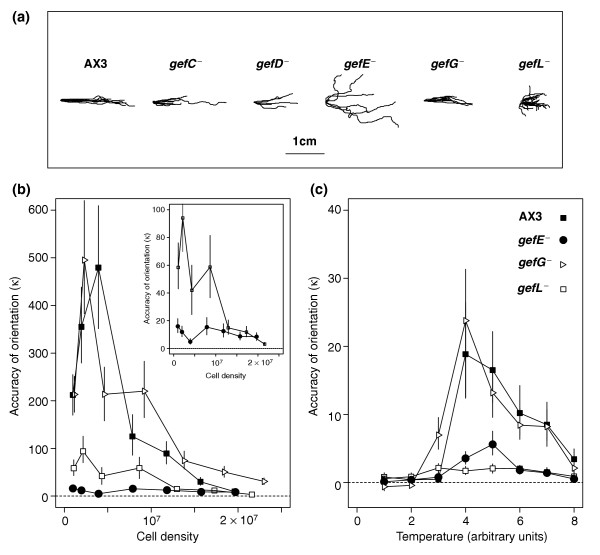
Phototaxis of *gef *mutant slugs. **(a) **Traces of slug trails from slugs migrating towards a light source to the right of the figure. Slugs migrated on charcoal agar plates; trails were transferred to white filters and stained with Coomassie blue. **(b) **Accuracy of slug phototaxis at different starting cell densities. Different quantities of cells were deposited on a charcoal agarose plate and allowed to move for 48 h. Trails were visualized as above, and phototactic efficiency was measured as described in Materials and methods. The inset shows data for *gef*E and *gef*L mutants replotted with different axes to reveal significant, if diminished, phototaxis in each case. Other mutants behaved apparently normally (data not shown). **(c) **Dependence of slug thermotaxis on temperature. Cells were plated and thermotaxis measured as described in Materials and methods. Other mutants behaved apparently normally (data not shown).

While testing the late development of mutants, we observed an apparent lack of slug phototaxis in *gefE *mutants, despite morphologically normal slugs and fruiting bodies. We therefore assessed slug phototaxis in each of the mutants apart from *gefB*, which does not form slugs. Figure 6a shows the trails from a sample of slugs migrating towards a lateral light source. Mutants in *gefC*, *gefD*, *gefG *and *gefK *(not shown) exhibit normal phototaxis, but *gefE *and *gefL *mutants are plainly aberrant. The problems with the two strains appear to be different. *gefE *slugs migrate similar distances to the wild type, but far less accurately towards the light source, whereas *gefL *slugs are both less accurate and migrate shorter distances, a phenotype that is frequently observed in phototaxis mutants [44].

A more detailed analysis of slug photo- and thermotaxis is shown in Figure 6b and 6c. Both *gefE *and *gefL *mutant slugs are clearly poor in phototaxis assays at all cell densities, with *gefE *mutants the worst affected (Figure 6b, inset) - *gefE *mutant slugs are at least 20 times less phototactically accurate than wild type, though some orientation is clearly visible (Figure 5a) and measurable (Figure 6b, inset). This behavior is strikingly reminiscent of the phenotype of *ras*D mutants, which also move normally but with greatly reduced accuracy [25]. Taken together with the expression pattern of *gefE*, which closely resembles that of *rasD*, this suggests that RasGEFE is the GEF that causes the most RasD activation during phototaxis.

Unusually for slug phototaxis mutants, the *gefE *and *gefL *mutant slugs are also affected in thermotaxis, although the severity of the phenotypes is reversed. Slugs from both mutant strains show diminished but measurable thermotaxis, but *gefL *mutant slugs seem particularly insensitive to temperature gradients (Figure 6c).

## Discussion

We initiated this work to assess whether the large number of RasGEFs in *Dictyostelium *were functionally redundant, or whether each had a discrete function. Functional redundancy would be caused by groups of RasGEFs having shared or poorly differentiated functions. In particular, one model for RasGEF action suggests that Ras signaling works as a complex network in which specific signals can be transduced by multiple RasGEFs, each of which can activate multiple different Ras proteins. If this model were correct, deletion of any one RasGEF (or indeed Ras) would cause very slight effects, with double and multiple mutants causing progressively more significant deficiencies in a range of different Ras pathways.

The work described in this paper, in agreement with previous work from our labs (for example [42]) and others [43], suggests that RasGEFs have relatively precisely defined roles. Of the RasGEF genes that have now been disrupted, six out of ten had clear phenotypes. We presume that a more complete study of the minute details of the life cycle would also reveal phenotypes in some of the remaining four. This would not be predicted if genetic redundancy was the rule. These results are somewhat distorted, because the RasGEFs were in general named in the order in which they were isolated. The majority of the genes we disrupted were identified relatively early in the lifetime of the cDNA project in Tsukuba, Japan, and therefore tend to be expressed at reasonably high levels. Later genes were identified by screening sequences provided by the genome project and are therefore likely to be either expressed at lower levels or under non-standard conditions, for example, environmental responses that are not seen under laboratory conditions. Even with this caution in mind, though, the clear phenotypes suggest that *Dictyostelium *uses relatively simple Ras pathways.

This is further supported by the similarities between specific Ras and GEF mutants. As previously described, Mutants in *gefB *and *rasS *behave similarly [23,29]. Likewise, in this work we show that *gefE *resembles *rasD *in both mutant phenotype and expression pattern. The *aimless*/*gefA *phenotype is also very similar to that of *rasC *[24,28], although one paper suggests a connection between *rasG*, *gefA *and the effector protein RIP3 [45]. Again, this suggests that *Dictyostelium *Ras pathways are relatively linear, with each GEF in general acting on a single Ras protein, rather than the networks that some might have expected.

This, of course, raises the question of what role is played by the other GEFs we describe. The *rasB *gene has not yet been disrupted, despite multiple attempts. It is thus not currently possible to discern its biological role. No RasGEF genes have been found to cause phenotypes that resemble those of *rasG *mutants. Finally, at least two other Ras-family proteins (RasX and Rap1) are likely to be activated by RasGEF family members [22]. In mammalian cells, the catalytic domains of RapGEFs are indistinguishable from RasGEFs, and frequently contain no obvious signaling domains much like several of the GEF genes described in this paper. We have connected three of the RasGEFs with specific Ras proteins, leaving 22 RasGEFs to couple to the smaller number of remaining Ras family members. This inequality implies that each Ras protein is likely to be activated by multiple RasGEFs, perhaps explaining the slight phenotypes seen in the unpaired RasGEF mutants.

We have not studied the *gflA*, *gflB*, *gflC *and *gflD *genes, which encode carboxy-terminal RasGEF homology domains without RasGEF amino-terminal domains, and might therefore be expected to be specific for Ras-related proteins that are not part of the Ras family proper. It is not entirely clear what this might mean for *Dictyostelium*. Two Ras proteins (RasG and RasD) are most similar to the canonical mammalian Ras families, and others (RasB, RasC, the as-yet unpublished RasX, and RasS) are less closely related to mammalian Ras while still plainly being part of the Ras family [22]. We suspect from the phenotypes of the *gefB *mutants that RasGEFB acts directly upon RasS. This would imply that the *gfl *genes act on more distant relatives of canonical Ras than RasS, which would be characterized as unusual small GTPases (for example the RasX family or RsmA-K [22]) rather than members of the Ras family proper.

Above all, the complexity in Ras pathways in *Dictyostelium *is unexpected for a relatively simple organism with no receptor tyrosine kinases [27]. It might be that the limited range of receptors was a driving force for diversification of Ras signaling during evolution, or it might be that a large number of RasGEFs are highly specialized for specific subsets of some complex role (detecting and integrating starvation signals and quorum factors, for example). A complete understanding will require further analysis of RasGEF genes, but above all a better knowledge of the range of signals that *Dictyostelium *cells use in their normal biological context.

## Conclusion

The clear suggestion from our work is that the unexpectedly large number of *Dictyostelium *RasGEFs derive from an unusually diverse range of inputs to Ras pathways, rather than large-scale redundancy or multiple pseudogenes following gene duplications. Identifying the input signals, and the mechanisms by which they connect to RasGEF activation, will be a major future challenge for *Dictyostelium *biology.

## Materials and methods

### Identification of genes encoding RasGEFs

Known and previously identified rasGEF genes were used to perform TBLASTN searches against the whole dataset generated by the *Dictyostelium *Sequencing Consortium [46-49]. In addition, IPRscan results for the predicted proteome of the previously published chromosome 2 [50] were screened for motifs IPR001895 (RasGEF) and IPR000651 (RasGEF amino-terminal [51]). In addition, we applied hmmsearch (HMMer package [52]) to scan the protein-translated pre-draft genome assembly (ORFs expected to be about 30 amino acids) for Pfam motifs PF00617 (RasGEF) and PF00618 (RasGEF amino-terminal). Contig sequences generated as described in [53] were extended and verified as described [50] to obtain full and high-quality coverage of the genes. Gene models were predicted using geneid [54]. The *Dictyostelium *genome is housed and curated at dictyBase [32].

### Domain analysis

The predicted complete amino-acid sequences were analyzed using the SMART program at the SMART website in Heidelberg [34]. In initial searches, borderline matches and matches from other libraries were included to ensure that important genes or domains were not excluded (in particular, several of the RasGEF amino-terminal domains are close to the borderline of significance, on either side). Images were copied directly from the SMART website.

### Cell growth and development

*D. discoideum *AX3 and AX2 cells were either grown axenically in HL5 medium or on a bacterial food source at 22°C [55]. For bacterially grown cells, SM agar plates were inoculated with 10^5^-10^6 ^*Dictyostelium *cells plus a suspension of *Klebsiella *in LB. To follow differentiation, cells growing exponentially from bacterial plates or axenic growth media were washed three times in KK2 (16.5 mM KH_2_PO_4_, 3.8 mM K_2_HPO_4 _pH 6.0) and plated on KK2 agar or nitrocellulose filters (Millipore). Transformation was performed by a modification of Howard *et al*. [56]; briefly, cells growing exponentially were mixed with 25 μg of linearized DNA and electroporated in a BioRad gene pulser at 1.0 or 1.1 V, 3 μF with a 5-ohm resistance in series. After 10 min incubation on ice, cells were placed at 22°C for 15 sec in the presence of 2 μl healing solution (100 mM MgCl_2_, 100 mM CaCl_2_) and then HL-5 added. Blasticidin-S (ICN) or G418 (Calbiochem) (10 μg/ml) was added 24 h after electroporation. After 7-8 days antibiotic selection, transformants were cloned on lawns of *Klebsiella *growing on SM agar.

### Gene-disruption constructs

Genes were disrupted using the blasticidin cassette from pRHI100, a derivative of pBrsΔBam [57] with additional restriction sites. Clones for each RasGEF kindly provided by the Japanese cDNA database were cut with an appropriate restriction enzyme to generate a site near the middle of the cDNA, and the Bsr gene was inserted into the gap using a rapid ligation kit (NEB). The knockout construct was cut out of the vector (usually using *Sal*I and *Not*I) and electroporated into axenic *Dictyostelium *as described above. Disrupted genes were identified using Southern blots with the entire cDNA used as a probe.

### Northern blotting and RT-PCR

Development of *D. discoideum *cells, isolation of RNA at different time points of development (0, 4, 8, 12, 14 and 18 h) and Northern blotting was performed as described [58]. RNA (5 μg) from each time point was reverse transcribed with random prime hexanucleotides using M-MLV RNase H(-) reverse transcriptase according to the manufacturer's protocol (Promega). 1 μl of a 1:10 dilution of the cDNA was used in PCR reactions with gene-specific primers for the different RasGEFs. Primers of 30 bases for product sizes of ~500 bp were selected with the program GeneFisher [59]. PCR was performed at 94°C for 60 sec (denaturation), 55°C (RasGEFG, O, P and S) or 60°C (all others) for 45 sec (annealing), 68°C for 60 sec (elongation) and with 25, 30 (RasGEFO and P) or 40 (RasGEFG and S) cycles. Reactions were separated on agarose gels. RT-PCR and Northern blot results were scored according to the key in Table 1.

### Phototaxis and thermotaxis

Qualitative phototaxis tests were performed as described previously [44] by using sterile spatula-style toothpicks to transfer cells to charcoal agar plates from the edges of colonies growing on *Klebsiella aerogenes *lawns. Phototaxis was scored after 48 h incubation at 21°C with a lateral light source. For quantitative phototaxis experiments, washed amebae were inoculated onto the centers of charcoal agarose plates (pH 6.5) at various densities and incubated with a lateral light source for 48 h at 21°C. For quantitative thermotaxis experiments, washed amebae were inoculated onto the centers of water agarose plates (~2.4 × 10^6 ^cells/cm^2^) and incubated for 72 h in darkness on a heat bar producing a 0.2°C/cm temperature gradient at the agarose surface. Arbitrary temperature units correspond to a temperature range of 14°C (T1) to 28°C (T8), as measured at the center of plates in separate calibration experiments. Slug trails were transferred to PVC disks, stained with Coomassie Blue, and digitized. Slug orientation was analyzed using directional statistics [60].

## Figures and Tables

**Table 1:** Expression of *gef *genes during growth and development

	0 h	4 h	8 h	12 h	14 h	18 h
*gefA *(*aleA*)*	+++	+++	+++	ND	ND	ND
gefB*	+	+	++	++	++	+
gefC*	-	-	+++	+++	+++	++
gefD*	+	+	++	++	+	+
gefE*	-	-	-	++	+	+
gefF*	+	++	+	+	+	+
gefG*	-	-	-	+/-	+/-	-
gefH*	+++	+++	+++	+++	++	+
*gefI*^†^	+	++	++	+++	++	++
gefJ1/J2*	+	+	+	+	+	+
*gefK*^†^	+	++	++	+++	++	++
gefL*	++	++	++	ND	++	++
*gefM*^†^	++	++	++	+++	+++	++
*gefN*^†^	+	++	++	+++	++	++
*gefO*^†^	+/-	+/-	+/-	+	+/-	+/-
*gefP*^†^	+/-	+/-	+/-	+	+/-	+/-
*gefQ*^†^	++	++	+++	+++	++	++
*gefR*^†^	+	+	++	++	++	++
*gefS*^†^	+/-	+/-	+/-	+	+	+/-
*gefT*^†^	++	+++	+++	+++	++	+
*gefU*^†^	+	++	+++	+++	++	+
*gefV*^†^	++	++	++	+++	++	++
*gefW*^†^	++	++	++	+++	++	++
*gefX*^†^	++	++	+++	+++	++	++
*gefY*^†^	++	++	+++	+++	++	++

A summary of results from Northern blots, RT-PCR and published data. GEFs are indicated in the left column and hours of development in the top row. *gefJ *is encoded by two separate but near-identical genes which cannot be differentiated at this level. *Northern Blot; ^†^RT-PCR. +++, strong band; ++, clear band; +, weak band; +/-, hardly detectable; -, no expression detected; ND, not done.
